# Angle Sensor Module for Vehicle Steering Device Based on Multi-Track Impulse Ring

**DOI:** 10.3390/s19030526

**Published:** 2019-01-27

**Authors:** Seong Tak Woo, Young Bin Park, Ju Hee Lee, Chun Soo Han, Sungdae Na, Ju Young Kim

**Affiliations:** 1 Gyeongbuk Institute of IT Convergence Industry Technology, Gyeongsan-si 38463, Korea; ybpark@gitc.or.kr (Y.B.P.); jhlee@gitc.or.kr (J.H.L.); jykim@gitc.or.kr (J.Y.K.); 2 SKF Sealing Solutions Korea Co., Ltd., Dalseong-Gun Daegu 49233, Korea; shunsoo.han@skfkorea.co.kr; 3 Department of Biomedical Engineering Center, Kyungpook National University Chil-gok Hospital, Daegu 41404, Korea; bluepoison14@knu.ac.kr

**Keywords:** EPSS (Electric Power Steering System), TAS module, multi-track encoder, self-driving Car, steering angle sensor

## Abstract

In step with the development of Industry 4.0, research on automatic operation technology and components related to automobiles is continuously being conducted. In particular, the torque angle sensor (TAS) module of the steering wheel system is considered to be a core technology owing to its precise angle, torque sensing, and high-speed signal processing. In the case of conventional TAS modules, in addition to the complicated gear structure, there is an error in angle detection due to the backlash between the main and sub-gear. In this paper, we propose a multi-track encoder-based vehicle steering system, which is incorporated with a TAS module structure that minimizes the number of components and the angle detection error of the module compared with existing TAS modules. We also fabricated and tested an angle detection signal processing board and evaluated it on a test stand. As a result, we could confirm its excellent performance of an average deviation of 0.4° and applicability to actual vehicles by evaluating its electromagnetic interference (EMI) environmental reliability. The ultimate goal of the TAS module is to detect the target steering angle with minimal computation by the steering or main electronic control unit (ECU) to meet the needs of the rapidly growing vehicle technology. The verified angle detection module can be applied to an actual steering system in accordance with the mentioned technical requirements.

## 1. Introduction

Recently, research on parts related to automobiles, such as LiDAR, ECU, and advanced steering control devices, has been advancing along with the development of autonomous vehicle technology [1,2,3,4,5,6,7,8,9,10]. In particular, the steering system plays an important role in timely control of the traveling direction and turning angle of a vehicle through precise angle detection and fast data processing as a key system that affects the stability and performance of the vehicle [11,12,13,14,15].

In addition, the torque angle sensor (TAS) module, which is a component of an electronic power steering system (EPSS) that improves the convenience and stability of operation, is mounted on the lower end of the steering wheel along with a steering angle sensor (SAS) and steering torque sensor (STS) to measure the torque applied to the steering shaft.

**Figure 1:**
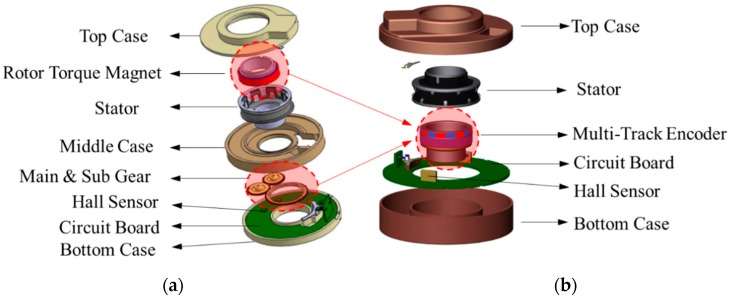
(**a**) Conventional torque angle sensor and (**b**) proposed torque angle sensor based on MTE schematic.

In this paper, we propose a multi-track encoder (MTE)-based TAS module that can downsize the module by simplifying its structure and has better angle detection resolution than the existing TAS modules. The angle detection signal processing module was developed and evaluated. The conceptual diagram of a conventional TAS module and TAS with the proposed structure is shown in Figure 1. The proposed TAS module can miniaturize the detector, and thus, the overall module by directly detecting the steering angle and torque through magnetic contactless sensing with the MTE, and it is possible to achieve better angular resolution.

In addition, we analyzed the angle detection process applied to the proposed structure through MTE through a MATLAB-based signal processing program. In particular, the inter-channel distance and pattern are very important factors in the MTE application of the TAS module. According to the uniformity of the MTE pattern, the difference signal of the two sine and cosine signals is different, and the angle detection data can be influenced.

In Figure 1, the existing TAS module performs angle detection and calculation through the gear ratio when the main and sub- gears rotate, and there is a disadvantage in that an angle detection error occurs owing to the backlash generated when the gears are engaged [15]. To overcome this drawback, the TAS module of the proposed structure can detect angles and torques according to the MTE rotation through a Hall sensor in a non-contact manner. Therefore, the proposed TAS module structure does not have backlash because it does not use gears, and angle detection with the MTE is possible even at high-speed rotation through the non-contact method. Generally, a conventional steering module has a detection speed limit of 2000–3000°/s imposed by gear rotation, but the TAS module structure proposed in this paper is not affected by speed [16]. In this study, we implemented the signal processing module with built-in MTE and digital Hall sensor to build the proposed TAS module, and analyzed the angle detection characteristics, which achieved better performance compared with the existing TAS model.

## 2. Element Technology

### 2.1. Fabrication of MTE

**Figure 2:**
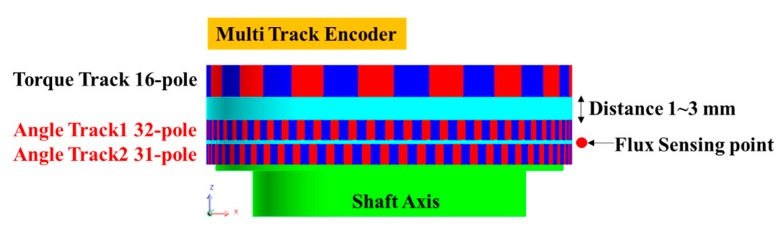
MTE design for finite element analysis.

MTEs represent one of the most important components in angle detection; hence, it is important to form a measurable stray magnetic field with rotation. In this study, for the MTE design with high magnetic field characteristics and optimized specifications, track spacing and material characteristics were analyzed using a finite element analysis program (Flux, CEDRAT). A double track for angle detection and single track for torque detection were designed as 32-pole, 31-pole, and 16-pole motors, respectively, and the characteristics were analyzed by changing the gap between torque and angle tracks from 1 mm to 3 mm. The MTE structure designed for the analysis is shown in Figure 2, and the results are shown in Table 1.

**Figure 3:**
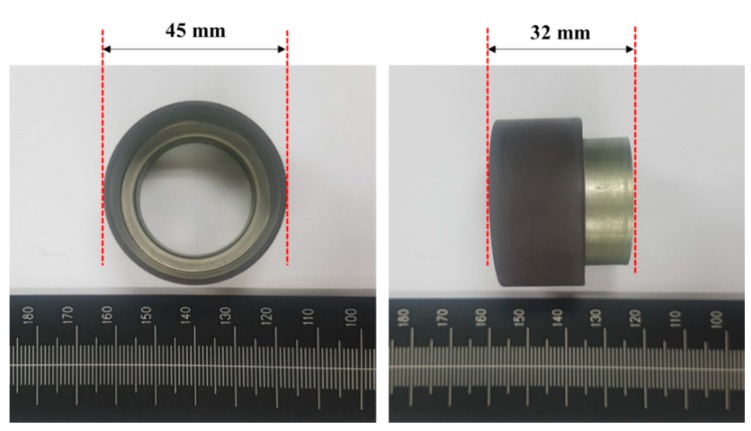
Photographs of the fabricated MTE.

The material of the encoder magnet used for the analysis was ferrite, and the magnetic field was measured at a distance of 1 mm vertically from the center of the two angle tracks. Table 1 shows a maximum difference of 6 Gauss for this interval, which does not have considerable effect when considering the minimum input sensitivity of 188 Gauss of the digital Hall sensor. However, if the distance between the torque track and angle track is narrower than 1–2 mm, the magnetic field formed along the direction of the polarity may be bridged and the signals cancel each other. Therefore, in this study, the MTE was fabricated with a distance of 3 mm between the torque and angle tracks, which was verified by the finite element analysis. An image of the MTE is shown in Figure 3.

Ferrite, Nitrile-butadiene Rubber (NBR), and SUS304 stainless steel were used in the finite element analysis model at volume ratios of 89.7, 10, and 0.03%, respectively. The outer diameter was 49 mm, the inner diameter was 45 mm, and its height was 20 mm. The addition of NBR series rubber, which acts as an external binder of the MTE, maximizes ferrite sintering and ease of fabrication, and SUS304 is added to optimize corrosion resistance and oxidation resistance. Based on the simulation results, the magnetic force at the center of the angle track (pore: 1 mm) of the MTE was approximately 260 Gauss, which was 30 Gauss lower than the simulation results in Table 1, and is 70 Gauss higher than the input sensitivity of the digital Hall sensor. The detection of data based on the MTE rotation is not an issue.

Ferrite, NBR rubber, and SUS304 stainless steel were used in the finite element analysis model at volume ratios of 89.7, 10, and 0.03%, respectively. The outer diameter was 49 mm, the inner diameter was 45 mm, and its height was 20 mm. The addition of NBR series rubber, which acts as an external binder of the MTE, maximizes ferrite sintering and ease of fabrication, and SUS304 is added to optimize corrosion resistance and oxidation resistance. Based on the simulation results, the magnetic force at the center of the angle track (pore: 1 mm) of the MTE was approximately 260 Gauss, which was 30 Gauss lower than the simulation results in Table 1, and is 70 Gauss higher than the input sensitivity of the digital Hall sensor. The detection of data based on the MTE rotation is not an issue.

### 2.2. Angle Calculation

**Figure 4:**
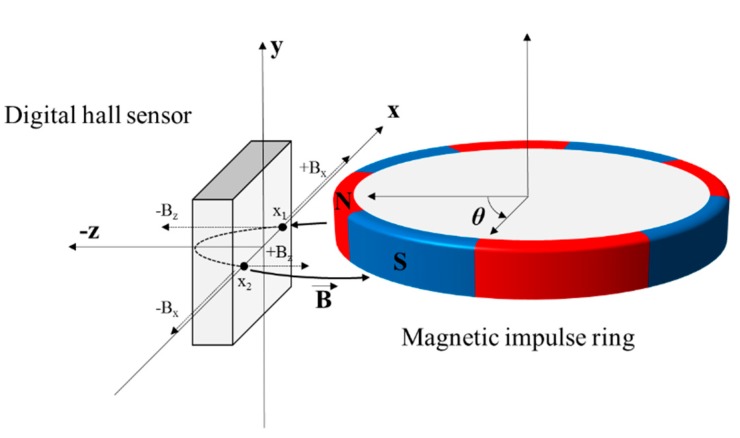
The principle of angle calculation between the hall sensor and magnetic impulse ring.

In general, the TAS module in the steering system of an automobile detects the absolute position by an output signal based on the ratio of the main and sub-gears in the apparatus through an arctangent calculation. The operation is relatively simple to implement, and processing time is short [17,18]. The basic formula of the arctan operation is as follows:

The magnetic flux characteristics of the Hall sensor are shown in Figure 4. A current-carrying conductive plate crossed by a magnetic field perpendicular to the plane of the hall-plate develops a crossing potential voltage. This hall-effects is described by the Lorentz force. This is because the electron that moves the magnetic field generates a force as shown in Equation (1).

(1) F=qv×B

where *F* is the resulting force, *q* is the electrical charge of the electron, *v* is the velocity of motion and *B* is the magnetic field. The track of the electron changes because of the resulting force, developing a potential voltage across the plate shown in Equation (2).

(2) $$V_{H}=\frac{IB_{vertical}}{\rho_{n}qt}$$

where *V_H_* is the hall voltage, *I* is the current passing through the plate, *B_vertical_* is the perpendicular magnetic field, *n* is the number of carriers per volume, *q* is the charge and *t* is the thickness of the plate. The magnetic force characteristics acting on the plate points *x*_1_ and *x*_2_ in the Hall sensor are expressed by Equation (3). Furthermore, *B_x_* and *B_z_* acting perpendicularly to the plane of the Hall sensor are expressed by Equation (4).

(3) $$x_{1}=S_{x}B_{x}+S_{z}B_{z},x_{2}=-S_{x}B_{x}+S_{z}B_{z}$$

(4) $$B_{x}=\frac{\left(x_{1}-x_{2}\right)}{2S_{x}},B_{z}=\frac{\left(x_{1}+x_{2}\right)}{2S_{z}}$$

where *S_x_*, *S_z_* are the respective sensitivity of Hall sensor for the *x* and *z* axis, it is generally seen that the sensitivity is the same. As the impulse ring is rotated, the Hall sensor is detecting the vector of the magnetic field from the impulse ring and generates signals *Vx* and *Vz* that are proportional to the Sin and Cosine signals respectively as shown in Equation (5).

(5) $$V_{x}=S_{x}\times B_{x}\times \cos\theta ,V_{z}=S_{z}\times B_{z}\times \sin\theta$$

The absolute angle *θ* can be derived from calculating the arctan of the voltages *V_x_* and *V_z_* as shown in the following Equations (6) and (7) [19,20].

(6) $$\theta_{1}=90{}^{\circ}+tan^{-1}\left(\frac{V_{x}}{V_{z}}\right)if\;V_{z}\geq 0$$

(7) $$\theta_{2}=270{}^{\circ}+tan^{-1}\left(\frac{V_{x}}{V_{z}}\right)if\;V_{z}<0$$

**Figure 5:**
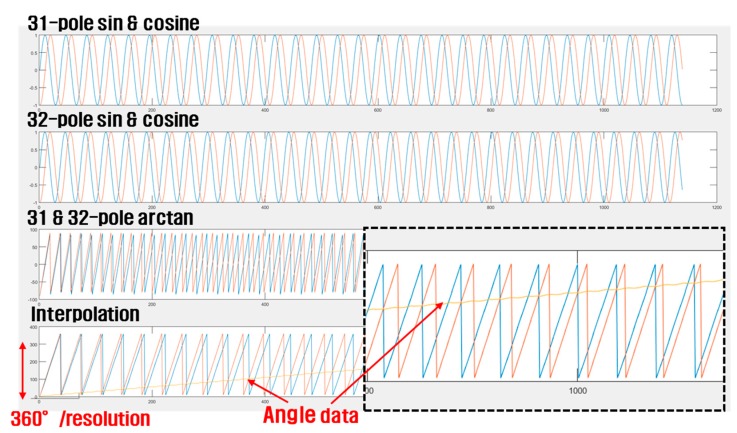
Results of angle detection simulation using MATLAB.

In Equations (6) and (7), *V_x_* and *V_z_* are cosine and sine signals measured through the pole-pair MTE, respectively. In this study, based on Equations (6) and (7), the angle data detected when the MTE is designed with 31 and 32 pole pairs rotated by 360° is qualitatively simulated using MATLAB software. The results are shown in Figure 5.

The sine signals provide angular data, which are calculated according to a positive or negative number and follow a triangular saw tooth waveform as shown in the third graph in Figure 4. The *y*-axis angle data (yellow line) are derived from the difference of each data point measured and calculated from the 31 and 32 pole pairs, as shown in the last graph in Figure 5.

### 2.3. Fabrication of Angle Detection Circuit

**Figure 6:**
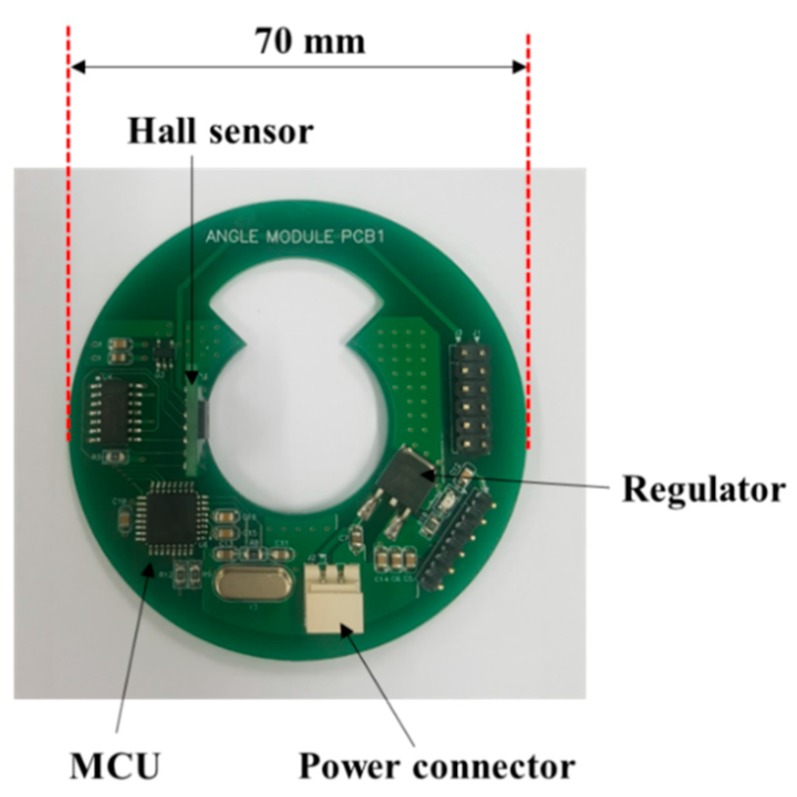
Fabricated angle sensor module.

The angle detection module was fabricated for comparison with the simulated angle value through the law data of the MTE pole pair, and the implemented board is shown in Figure 6.

The magnetic flux according to the rotation of the MTE is measured through a digital Hall sensor (IC-MU, IC-Haus). The angle binary data converted through the built-in arctan operation protocol is sent to the signal processor MCU (ATmega328, Atmel, San Jose, CA, USA) and is then calculated as an angle value. The digital Hall sensor is set to a resolution of 15 bits, which has an angular resolution of approximately 0.01°. Furthermore, the circuit was designed considering a standard shaft axis so that it could be applied to real automobiles, and it was designed to be driven by the standard automobile battery input voltage of 12 V.

### 2.4. Angle Sensor Module Experiments

**Figure 7:**
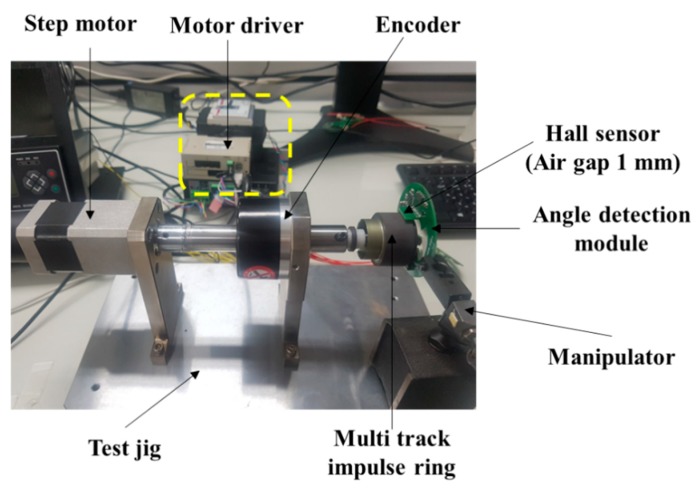
Test stand and experimental setup.

In this study, a test stand was fabricated and evaluated for the quantitative evaluation of the proposed angle detection module. The test stand is shown in Figure 7.

**Figure 8:**
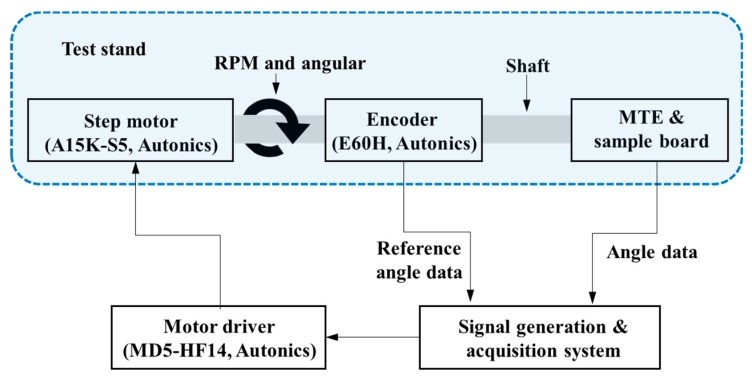
Experimental environment block diagram.

Conventional automotive steering modules have an angular deviation of about 0.6–1.0° [12,13,16]. The angle detection module implemented in this study has a maximum angular resolution of 0.01°. To evaluate the implemented module, the test stand was fabricated and verified through a step motor and encoder with maximum resolution of 0.01°. The angular data measured by the encoder and the manufactured angle detection module were compared by running the motor at intervals of 10°. The test environment block diagram of the angle detection module is shown in Figure 8. The step motor (A15K-S5, Autonics, Pusan, Korea) used in the evaluation is set to be driven at 0.01° resolution, and the encoder for reference angle detection (E60H-20-8192, Autonics, Pusan, Korea) has a resolution of 0.01°.

Also, to analyze the characteristics according to the rotation speed of the proposed module, the angle detection performance was evaluated at 3000°/s, 4000°/s, and 5000°/s. The TAS module 3000°/s has a speed of 500 rpm when converted to rpm as a unit representing the maximum rotation angle per second. (ex. 3000°/s = 500 rpm, 4000°/s = 666 rpm, 5000°/s = 833 rpm, 6000°/s = 1000 rpm). In general, conventional commercial TAS modules have a rotation speed of 2000–3000°/s. On the other hand, unlike the existing TAS module, the proposed module is not in the state of being engaged with sub-gears, so it is a floating type and has a great advantage in steering wheel rotation speed.

A PC-based signal generating and collecting system and motor driver (MD5-HF14, Autonics) were used to drive the pulse-width modulated (PWM) signal using a stepping motor, and the shaft was rotated according to the set angle with the motor running. At this time, a hollow encoder for detecting the reference angle, built-in MTE, and digital Hall sensor composing the angle detection module implemented at a distance of 1 mm between the MTE and air gap were placed on the shaft axis. The angle data of rotation of the shaft were then obtained through the reference angle detection encoder and the manufactured angle detection module.

## 3. Experimental Results

### 3.1. Evaluation of Angle Detection

**Figure 9:**
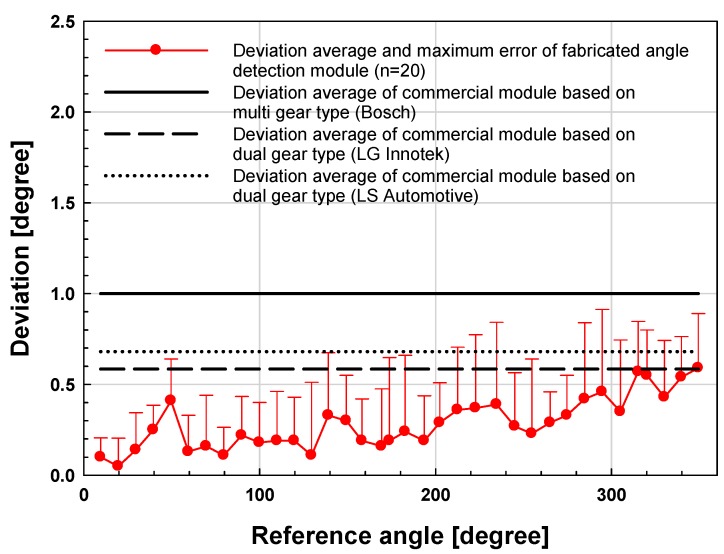
Results and comparison of the proposed angle sensor module and conventional module by test stand experiments versus reference angle.

The experimental results and conventional TAS module obtained through the test stand are shown in Figure 9. The reference angle was measured using a commercial encoder to verify the angle driven by the step motor and the angle was compared with the measurement data of the signal processing board for the implemented TAS module.

In Figure 9, deviations of the fabricated angle detection module and reference encoder data were measured for 0–360° in 10° intervals for angle detection. The measurements produced an average deviation of approximately 0.4° compared with the reference encoder. It shows improved characteristics over 5–8dB compared with commercial TAS modules. Most commercial TAS modules are multi-gear types with an average accuracy of about 0.6 to 1.0° [12,13,16].

**Figure 10:**
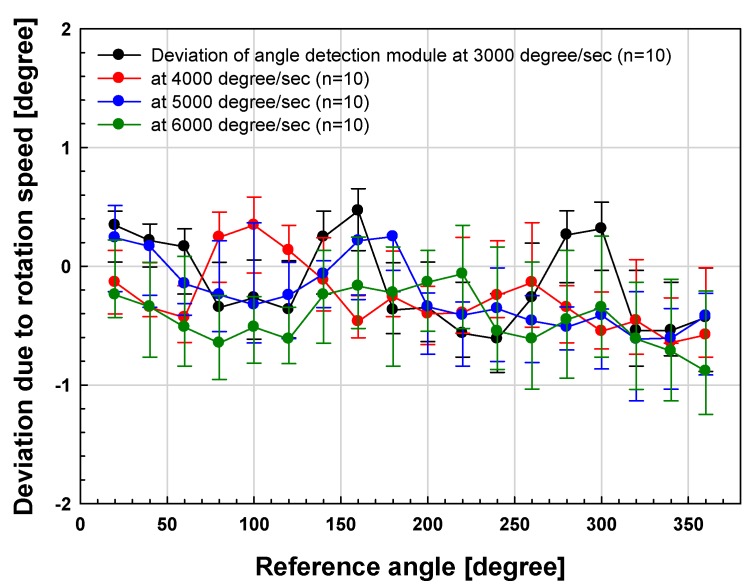
Deviation results of the proposed angle sensor module due to rotation speed.

**Figure 11:**
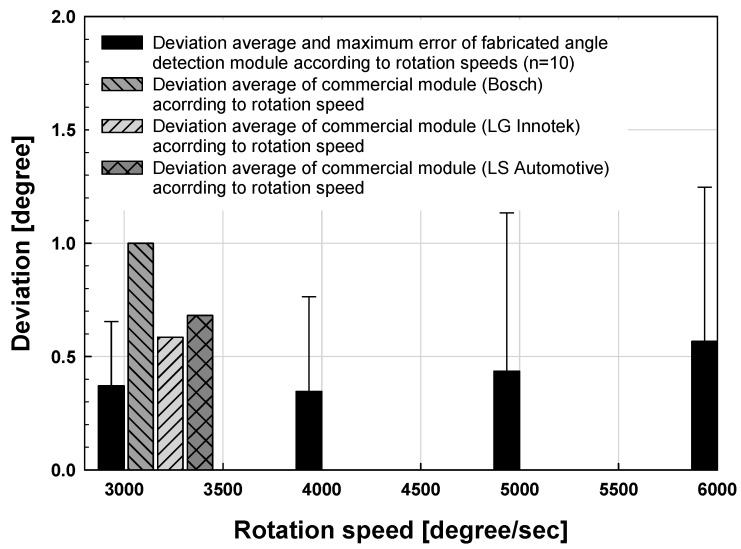
Deviation results of the proposed angle sensor module due to rotation speed.

In Figure 10 and Figure 11, the angle detection characteristics according to the rotational speed of the fabricated module were measured at intervals of 20° from 0 to 360°. As a result, the mean deviations were measured as 0.370°, 0.357°, 0.434° and 0.537° respectively at 3000°/s, 4000°/s, 5000°/s and 6000°/s, and the deviation increases in the section where the rotation speed is high. However, the maximum rotational speed characteristic of the conventional commercial TAS module is 3000°/s, and the proposed module has a relatively stable angle detection characteristic of about 0.5° standard deviation up to 6000°/s. Conventional TAS modules are multi-gear type gears. Therefore, it has a limitation on the rotation speed, and the performance is usually 2000 to 3000°/s. In particular, it causes a backlash phenomenon caused by gears, which can affect accuracy.

### 3.2. EMI Evaluation of Angle Sensor Module

**Figure 12:**
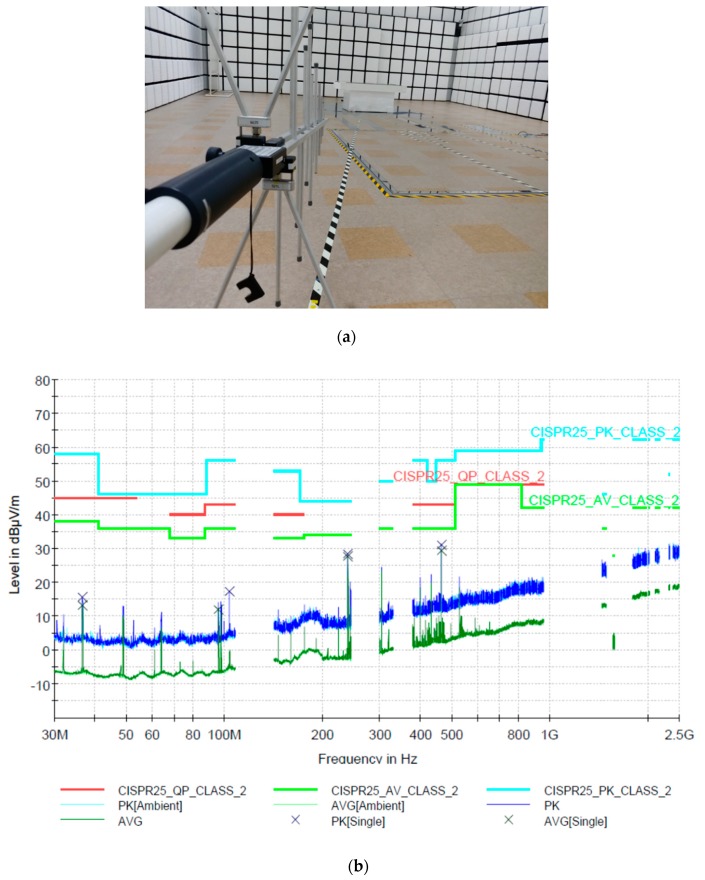

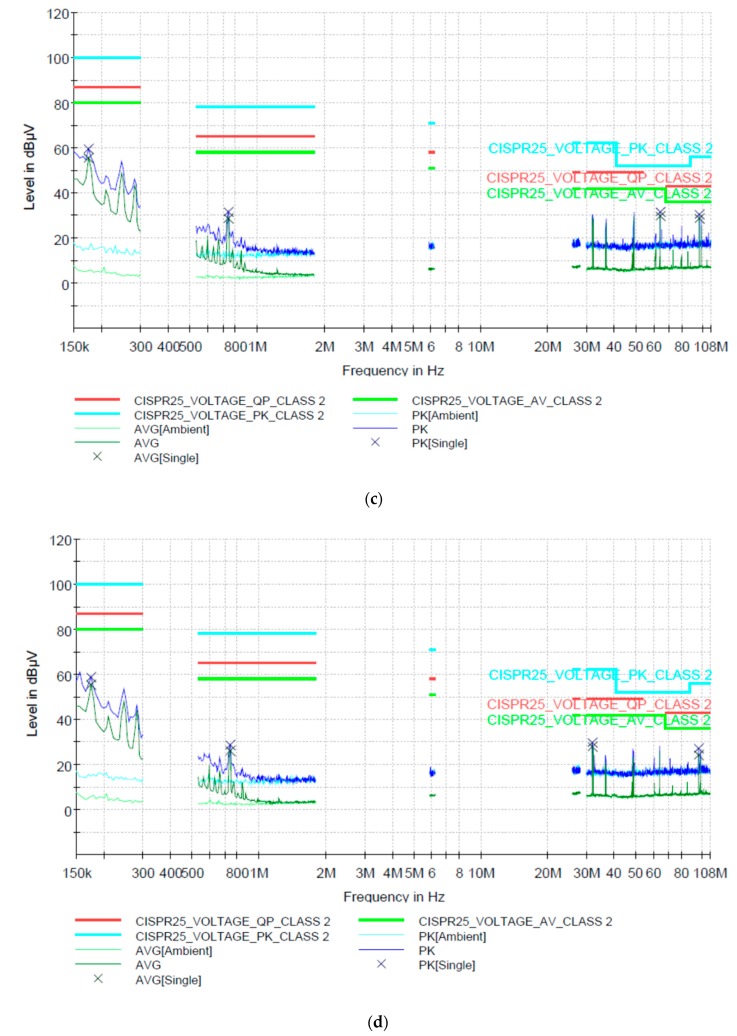
(**a**) EMI experimental environment, (**b**) EMI radiation emission (RE) characteristics of angle module about the horizontal and vertical axes, (**c**) EMI conducted emission (CE) characteristics for the ground line of the module, and (**d**) for the power line of the module.

To evaluate the environmental reliability of the angle detection board for the implemented TAS module, an electromagnetic radiation test was performed according to the CISPR25 standard for electronic parts. The experimental environment and measured test results are shown in Figure 12.

In general, electromagnetic interference (EMI) certification for automotive components is essential and complies with the CISPR25 standard. An automotive module can emit electromagnetic waves into the line or module itself connected to it during operation, which can have an electrical effect on the user or other components. Therefore, to regulate this problem to a certain level or less, EMI certification is performed. In the case of radiated tests, it is classified into the conducted emission (CE) emitted through the line of module and the radiation emission (RE) test, which measures the emission from the module itself. An electromagnetic radiation test was carried out in a sealed electromagnetic shielded room at an authorized testing laboratory, and the measuring distance was 10 m from the implemented module. When the operating voltage of 12V or 5V is applied, the CE measures electromagnetic waves through the line of module, it has 80, 58, and 40 dBuV standards in the range of 0.15 to 0.3 MHz, 0.5 to 2 MHz, and 25 to 108 MHz, respectively. In the CE test of the fabricated angle detection module, the average was measured to be 20–30 dBuV, which is in accordance with the standard. The RE emitted by the module itself has standard values of 35, 40 and 45 dBuV/m in the range of 0.03 to 0.25 GHz, 0.3 to 1 GHz and 1.5 to 2.5 GHz, respectively. In the RE test of the proposed module, the average was measured to be 10–25 dBuV, which is in accordance with the standard. The test measurements were less than 40 dBuV/m over the entire test band, which complied with the actual automobile electronic parts specification.

## 4. Discussion

The angle detection signal processing board implemented and evaluated in this study proved to achieve excellent angular resolution and detection accuracy based on experimental results. However, additional research and verification are required. First, it is necessary to optimize the torque detection function and specifications required for existing steering modules for automobiles. A detection method using a torque track in the designed MTE should be implemented and application of a linear Hall sensor standard is required to detect torque. Second, low power consumption and detailed standardization of automotive electronic components are required through application of automotive integrated circuits (ICs) based on the AEC-Q100 standard.

In the case of angular resolution, it is necessary to select the optimum range considering the power consumption of the signal processing module. The commercial TAS module has an angular resolution of 0.1–0.2°. In the case of the angle detection signal processing board implemented in this study, the angle detection deviation is approximately 0.4°, which is better than that of current commercial products. However, it is necessary to further improve performance by improving the test evaluation environment and through module optimization. In particular, the performance of the proposed angle detection module will allow for more accurate detection by optimizing the motor coupling structure of the test stand. This will lead to an accurate conclusion by minimizing the error on the vertical axis besides the rotary axis through the correction of the shaft center axis between the motor and the encoder. Therefore, the performance of the proposed angle detection module will allow for more accurate detection by optimizing the motor coupling structure of the test stand. This will lead to an accurate conclusion by minimizing the error on the vertical axis besides the rotary axis through the correction of the shaft center axis between the motor and the encoder. 

The correction of the rotation direction axis and the vertical axis in the module angle detection is a very important factor because the magnetic flux collected by the hall sensor changes due to the vertical axis error. This causes the nonlinearity of the hall sensor output signal and may further affect the arctan-based angle detection. In future research, it is necessary to supplement the performance of the proposed board with more precise verification by applying optical precision encoder in addition to the 0.01 resolution motor and encoder used in this experiment.

The proposed angle detection module has an average deviation characteristic of 0.4°, which is about 5~8 dB higher than conventional commercial products. Such a performance can be applied to an environment requiring precise angle detection, such as to an automatic parking system rather than to a steering angle detection function to implement an actual autonomous driving application. In particular, the active parking system requires high-precision steering angle information that can be used to optimize steering wheel turning by calculating the optimum steering angle by sensing the surrounding obstacles at a point set by the driver [21].

## 5. Conclusions

In this paper, we proposed an MTE-based TAS module which achieves better angular detection resolution than the existing main and sub-gear-based steering modules. The proposed MTE-based TAS module does not include the gear backlash error induced in existing commercial products as it does not use gears. The fabricated angle detection module uses a digital hall sensor and has a 15-bit resolution of about 0.01° and an average deviation of 0.4°. The experimental results show that the average deviation is 5-8 dB better than that of the commercial TAS module. Also, the proposed module showed an average deviation of about 0.35~0.57° at a rotation speed of 3000~6000°/s, through experiments, it was confirmed that the detection characteristics were satisfied at high speed. In addition, we conducted an EMI environment assessment based on vehicle specifications. Through the test results, we confirmed that it conforms to electromagnetic wave standards in all directions. 

In this paper, we believe that the qualified angle detection module can be used in the rapidly growing market for automotive steering parts.

## Figures and Tables

**Table 1:** Magnetic flux of the MTE according to distance between angle and torque tracks.

Distance [mm]	Magnetic Flux [Gauss]
1	295.8
2	292.9
3	289.8
